# Comments and Illustrations of the European Federation of Societies for Ultrasound in Medicine (EFSUMB) Guidelines: Rare Malignant Pulmonal and Pleural Tumors: Primary Pulmonary Sarcoma and Mesothelioma, Imaging Features on Transthoracic Ultrasound

**DOI:** 10.3390/diagnostics14202339

**Published:** 2024-10-21

**Authors:** Kathleen Möller, Florian Dietz, Michael Ludwig, Stephan Eisenmann, Christian Görg, Ehsan Safai Zadeh, Wolfgang Blank, Christian Jenssen, Veronika Vetchy, Burkhard Möller, Christoph Frank Dietrich

**Affiliations:** 1 Medical Department I/Gastroenterology, SANA Hospital Lichtenberg, 10365 Berlin, Germany; k.moeller@live.de; 2 Department General Internal Medicine (DAIM), Hospitals Hirslanden Bern Beau Site, Salem and Permanence, 3013 Bern, Switzerland; f.dietz@hotmail.com; 3 Department for Internal Medicine, Hospital of the German Armed Forces, 10115 Berlin, Germany; michael6ludwig@bundeswehr.org; 4 Department of Internal Medicine/Respiratory Medicine, University Hospital Halle, 06120 Halle (Saale), Germany; stephan.eisenmann@uk-halle.de; 5 Interdisciplinary Center of Ultrasound Diagnostics, Gastroenterology, Endocrinology, Metabolism and Clinical Infectiology, University Hospital Giessen and Marburg, Philipp University of Marburg, Baldingerstraße 10, 35037 Marburg, Germany; goergc53@gmail.com; 6 Department of Biomedical Imaging and Image-Guided Therapy, Medical University of Vienna, 1090 Vienna, Austria; ehsan_sz@yahoo.de (E.S.Z.); veronika.vetchy@meduniwien.ac.at (V.V.); 7 Klinikum am Steinenberg Reutlingen, Medizinische Klinik I, 72764 Reutlingen, Germany; wolfgang.blank@icloud.com; 8 Department for Internal Medicine, Krankenhaus Märkisch Oderland, 15344 Strausberg, Germany; c.jenssen@khmol.de; 9 Brandenburg Institute for Clinical Ultrasound (BICUS), Brandenburg Medical University, 16816 Neuruppin, Germany; 10 Department of Rheumatology and Immunology, Bern University Hospital, University of Bern, 3010 Bern, Switzerland; burkhard.moeller@insel.ch

**Keywords:** contrast-enhanced ultrasound, diagnosis, lung ultrasound, imaging, respiratory medicine, advances

## Abstract

Primary pulmonary sarcoma and mesothelioma are rare malignancies. The review article discusses the appearance of these tumors in B-mode ultrasound (US), color Doppler ultrasound and contrast-enhanced ultrasound (CEUS). In particular, the article is intended to inspire the examination of thoracic wall tumors and pleural masses with the possibilities of ultrasonography and to obtain histologically evaluable material using US or CEUS-guided sampling.

## 1. Introduction

Thoracic wall, pleural, mediastinal and lung ultrasound (US) with special consideration of the diaphragm has become established in daily medical routines, and continuous medical education (CME) review articles that have been updating the literature have been published recently [1,2,3,4]. In addition to the typical normal findings in adults, special features in children have also been discussed [5,6,7]. The special features of congenital diseases were emphasized and illustrated.

Innovative US techniques including contrast-enhanced ultrasound (CEUS) have been introduced for vascular diseases but also for the characterization of pleural and lung tumors and diseases [1,8,9,10,11,12,13,14,15,16]. The description of the CEUS procedure was published by the World Federation for Ultrasound in Medicine and Biology (WFUMB) [12]. The aim of the presented paper is to illustrate the appearance of primary pulmonary sarcoma and mesothelioma on transthoracic ultrasound and CEUS.

Summarizing what has been written above, there is a lack of knowledge and published papers on the wide range and diversity of lung pathologies. This paper presents primary pulmonary sarcoma and mesothelioma and analyzes the existing literature on their appearance in transthoracic US and CEUS. The aim of the paper is to provide a review on the use of lung ultrasound in primary pulmonal sarcoma and mesothelioma.

### Contrast-Enhanced Ultrasound (CEUS) of the Lung and Pleura

The ventilated lung is a strong reflector and leads to a total reflection of the ultrasound. Typical artifacts are the A-lines or reverberations in a healthy lung. Under these conditions, no assessment of the lung tissue is possible. In some disease situations, comet-like B-lines occur. These also make lung parenchyma assessment impossible. We refer to the corresponding literature [1,17,18,19,20,21,22,23]. Transthoracic ultrasound can only identify and assess lesions or masses that are not covered by normal lung tissue and lung artifacts. These are usually pleural lesions, subpleural peripheral lung lesions, masses in the pleural effusion or lesions in atelectatic lungs. This requirement also applies to CEUS.

SonoVue^®^ (Lumason^®^) is an intravascular ultrasound contrast agent and remains in the vessels without entering the interstitium. Adverse effects during CEUS of the various organ systems are rare. In over 49,000 patients, adverse effects occurred in 0.088% of all patients, only 7 patients (0.014%) suffered from serious AEs and no fatal event was reported [24]. In a multicenter study with >463,000 patients, 0.001% serious adverse effects and 0.034% non-serious adverse effects occurred. The severe AEs were most common in vascular indications [25]. In more than 89,000 patients with 0.024% adverse effects, wheezing/bronchospasm occurred in one patient, anaphylactic reactions in 3 patients [26].

Application of SonoVue^®^ (Lumason^®^) for examination of the lungs and pleura is off-label. However, no adverse effects particularly attributable to this particular indication have been reported so far, especially no clinically relevant effects on pulmonary hemodynamic parameters in patients with elevated or normal mean pulmonary artery pressure and lung diseases [27]. Nevertheless, information on off-label use should be included in the informed consent. Lipid-based UCAs (SonoVue^®^ and Lumason^®^) are contraindicated in patients with known hypersensitivity to these UCAs, polyethylene glycol (PEG, macrogol) or products containing PEG in the past [28,29].

The lung has a dual arterial blood supply: the pulmonary arteries and bronchial arteries [30]. This provides interesting differential diagnostic possibilities. The pulmonary arterial system is responsible for gas exchange. The bronchial artery system supplies the bronchi, pulmonary vessels, alveoli, interstitial tissue and visceral pleura with nutrients. The parietal pleura is supplied by the intercostal arteries.

The blood supply pattern of the lung and the intravascular location of the ultrasound contrast agent (UCA) SonoVue^®^ provide important information as to whether a lesion is supplied from the pulmonary arterial system or the bronchial arterial system. A time of enhancement (TE) before organs with systemic vascularization (thoracic wall, spleen, or liver) is typical for pulmonary arterial vascular supply, whereas enhancement simultaneous with organs with systemic vascularization indicates bronchial arterial enhancement [31]. In a given clinical context, the contrast-enhancement of a lesion can therefore provide valuable information on the type of lesion. More than 90% of atelectatic lung tissue shows pulmonary artery enhancement [32]. In contrast, bronchial arterial enhancement of peripherally located masses can be an important indication of malignancy [33,34]. However, peripherally located inflammatory consolidations may also show bronchial-arterial enhancement [13].

A short TE on CEUS can be used to differentiate acute inflammatory pneumonia lesions and compression atelectasis from other types of lung lesions [35,36]. Görg et al. described delayed TE in 62% of patients with peripheral malignant lesions [36]. Further criteria for assessing a lesion are the extent of enhancement and the homogeneity or inhomogeneity of the enhancement [36]. Other CEUS indices, such as time to peak (TP), mean transit time (MTT), extent of peak (EP), area under the curve (AUC) and slope, did not differ significantly between patients with benign and malignant lung disease and were not helpful [35,37]. Other important applications of CEUS are the differentiation of lung abscesses [38,39], peripheral pulmonary infarction [13,14,40] and the performance of biopsies under CEUS guidance [33,35,41,42,43]. CEUS provides indications for the differentiation of benign and malignant pleural thickening and masses [9,44] and supports the differentiation of tumors in the atelectatic lung [32]. Since the parietal pleura receives its blood supply from the intercostal arteries, the enhancement is comparable to systemic arterial vascularization. The distinction between pulmonary and bronchial arterial supply does not apply to the parietal pleura.

## 2. Primary Pulmonary Sarcomas

Thoracic sarcomas include a variety of different histologic types that occur in the lung, mediastinum, pleura and chest wall. Angiosarcoma, leiomyosarcoma, rhabdomyosarcoma and the sarcomatoid variant of mesothelioma are the most common primary intrathoracic sarcomas. Ewing’s sarcoma, primitive neuroectodermal tumor, chondrosarcoma, malignant fibrous histiocytoma, osteosarcoma, synovial sarcoma and fibrosarcoma usually arise in the chest wall [45]. Thoracic sarcomas can manifest as nodular lung lesions, endobronchial and in the pulmonary arteries. They exhibit a wide spectrum of radiologic manifestations, including solitary pulmonary nodules, central endobronchial tumors and intraluminal masses within the pulmonary arteries. The histologic subtype is often indistinguishable on radiologic imaging. However, a mass with calcified matrix may indicate a chondrosarcoma or osteosarcoma, and a mass in the pulmonary artery may indicate a leiomyosarcoma, for example [45]. Most common sarcomas of the chest wall are chondrosarcoma, osteosarcoma, Ewing’s sarcoma (primitive neuroectodermal tumor), malignant fibrous histiocytoma and fibrosarcoma [46].

Most pleural tumors are metastases of various primary tumors from the entire body, almost all primary lung malignancies are bronchogenic carcinomas and the vast majority of sarcomas that affect the lungs are metastases.

Primary pulmonary and pleural sarcomas (PPS) are rare. This entity accounts for <0.5% of all malignant pulmonary tumors [47,48].

The most common primary pleural sarcomas are malignant fibrous histiocytoma (MFH), liposarcoma, synovial sarcoma and solitary fibrous tumor. Pleural manifestations of osteosarcoma, chondrosarcoma, malignant peripheral nerve sheath tumor (MPNST), Ewing’s sarcoma and myeloid sarcoma/chloroma are even rarer [46]. The typical imaging features are usually nonspecific. There is a pleural mass and possibly a pleural effusion [46].

A study by Keel et al. of 26 primary lung sarcomas included 7 malignant fibrous histiocytomas, 6 synovial sarcomas, 3 malignant peripheral nerve sheath tumors, 3 leiomyosarcomas, 2 each of angiosarcomas, intimal sarcomas and fibrosarcomas and a single case of epithelioid hemangioendothelioma [49]. The smallest tumor measured 9 mm and the largest tumor covered the entire hemithorax [49].

Pulmonary mesenchymal tumors with sarcomas are included in the world health organization (WHO) classification of Lung Tumors 2015 [50] with an update in 2021 [51]. A new WHO Classification of soft tissue tumors was published in 2020 [52]. The mesenchymal lung tumors according to the WHO classification with a focus on their diagnostic pathology, molecular pathogenesis and identified biomarkers for differential diagnoses are described by Hashimoto et al. [53].

The prognosis is poor. In a study of 45 PPS within a period of 21 years, the median survival time and 5-year survival rates of resected and unresected PPS were 39.6 months and 28.7% and 4.9 months and 7.8%, respectively [47]. The clinical symptoms and imaging were similar to those of non-small-cell lung carcinomas (NSCLC). Locoregional invasion and distant metastases were common [47]. Typical symptoms are dyspnea, cough, chest, shoulder or back pain [47,48,54]. The imaging methods of choice for examining the lesions are contrast-enhanced computed tomography (CECT), position emission tomography-CT (PET-CT) and magnet resonance imaging (MRI). The focal findings are usually extended in the images [54]. In a review of case series of primary pulmonary synovial sarcomas, these appeared on CT as solitary, large, well-circumscribed masses that were heterogeneously enhanced in contrast. The tumors were mostly larger than 7 cm. Calcifications were described [48].

Although the primary pulmonary sarcomas in the case reports are often extensive and extend to the thoracic wall, there are no descriptions of correlating ultrasound findings.

Tsetsou et al. included ultrasound in their examination concept. They described a large extraosseous primary pulmonary Ewing sarcoma on the thoracic wall. On radiologic imaging, the tumor occupied a large part of the right-sided thorax. On B-mode ultrasound, the tumor appeared as a predominantly hypoechoic mass with amorphous calcifications, with central branching and irregular echogenic foci. Color Doppler Imaging (CDI) showed a rich vascularization with multiple, tortuous arteries surrounding avascular areas. The ultrasound also showed that there were no signs of thoracic wall infiltration. Histologic confirmation was performed by US-guided sampling with an 18 G needle from the pulmonary mass and a concomitant liver manifestation [55]. PPES are usually described in radiological imaging as a large inhomogeneous tumor with cystic degeneration with displacement of adjacent structures [45].

Li et al. describe the ultrasound appearance in B-mode, (CDI) and CEUS of a thoracic wall metastasis and renal metastasis of a primary pulmonary synovial sarcoma [56]. The thoracic wall metastasis was round, markedly hypoechoic, with a clear regular border. On CDI, punctate blood flow signals were seen inside the lesion. On CEUS, the lesion showed arterial hyperenhancement with rapid wash-out [56].

**Figure 1:**
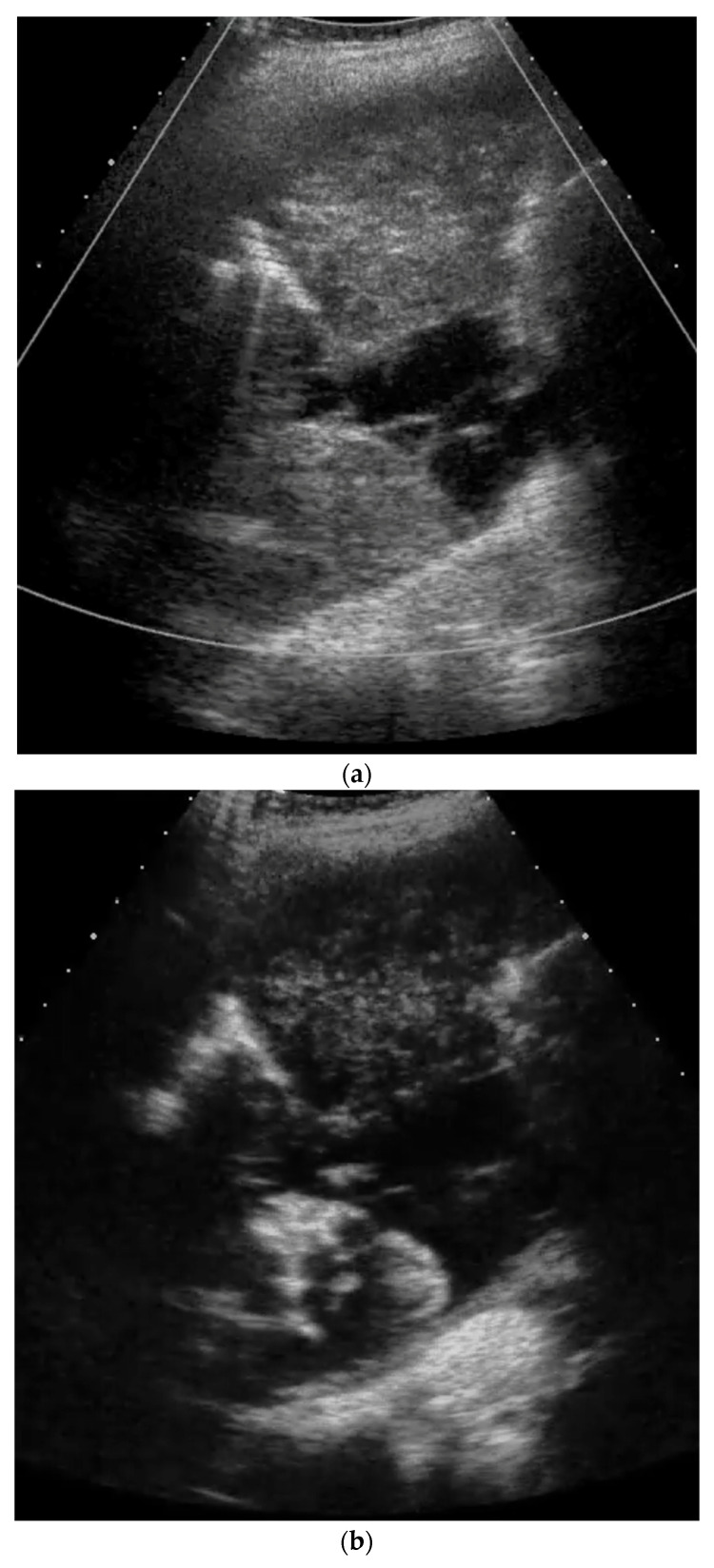

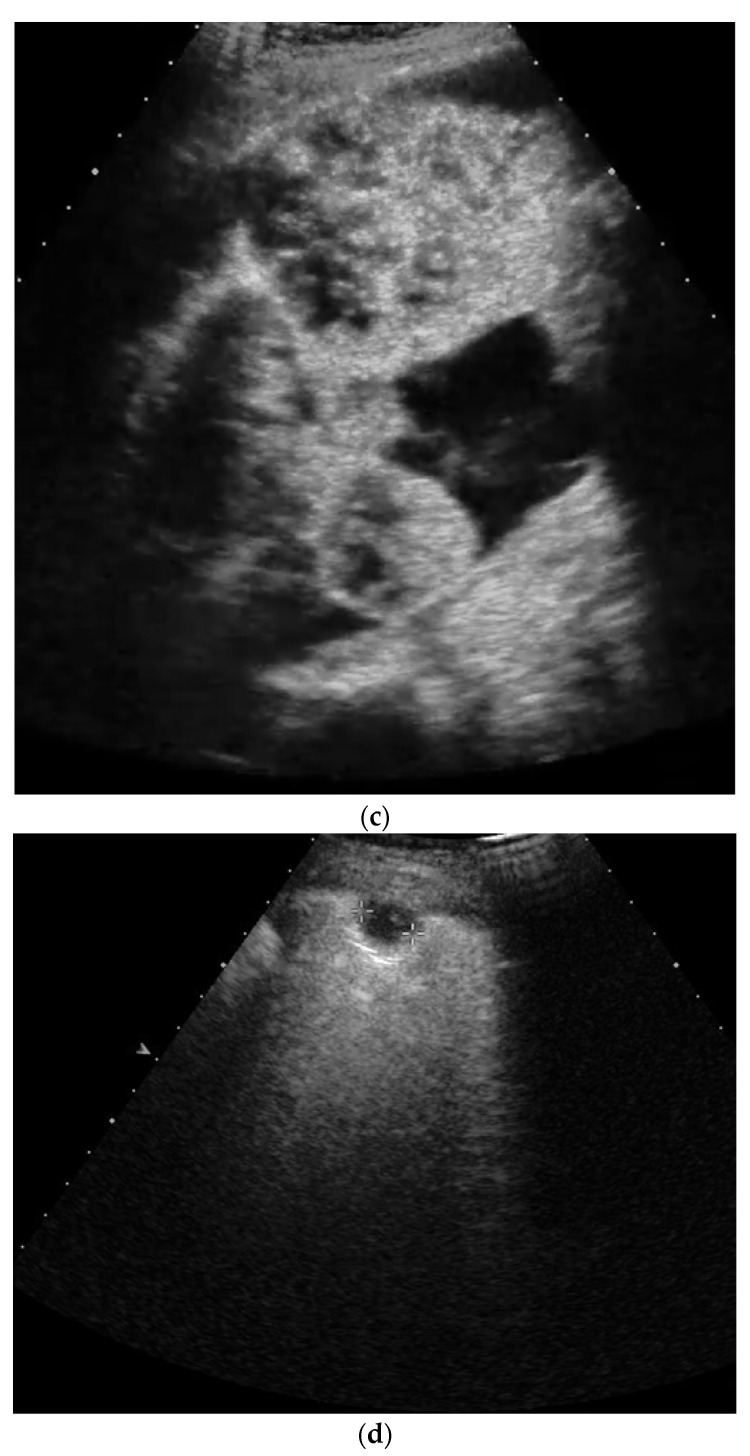
Primary sarcoma of the lung infiltrating almost the entire hemithorax proven by ultrasound-guided biopsy and histological evaluation with pleural metastasis. B mode ultrasound does not allow a clear differentiation between the lung parenchyma and the tumor (**a**). Contrast-enhanced ultrasound facilitates potential differentiation, because the atelectatic lung parenchyma is enhancing early via the pulmonary artery circulation (**b**), whereas the sarcoma shows later centripetal enhancement and non-enhancing (necrotic) areas (**c**). Multiple pleural metastases as well as a small pleural metastasis were found (**d**).

**Figure 2:**
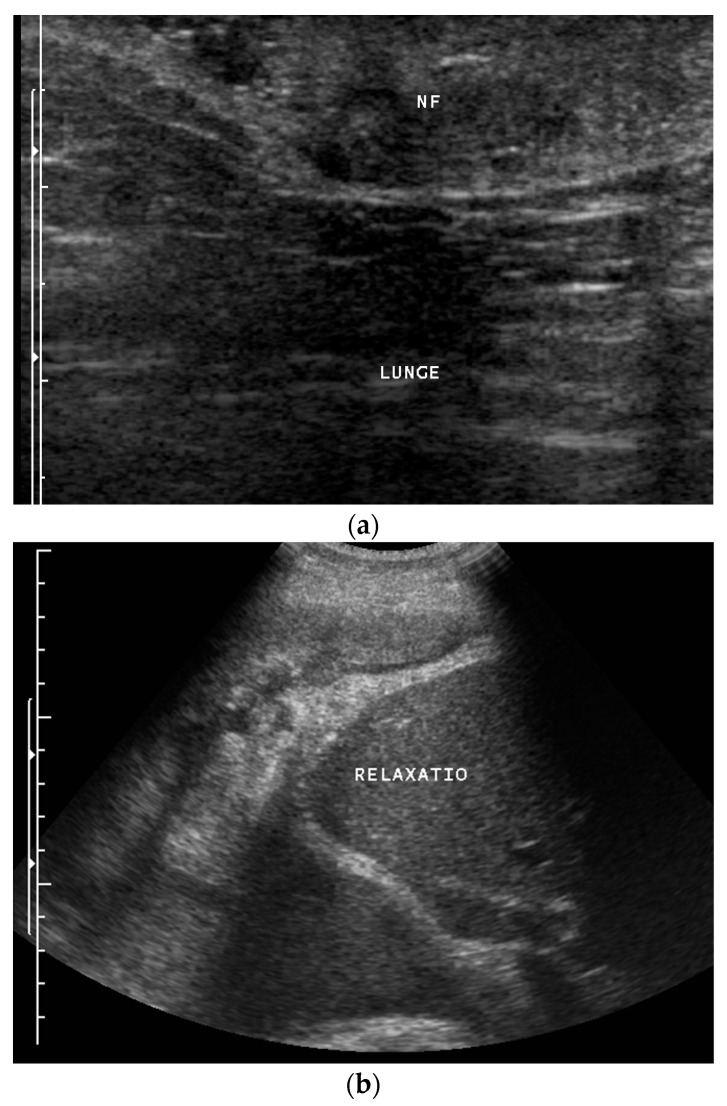
Potential neurofibroma of the thoracic wall (**a**) and biopsy-proven neurofibrosarcoma of the lung infiltrating almost the entire hemithorax (left and therefore upper part of the image) with paralysis of the diaphragm and bulging of the liver into the thorax (“Relaxatio”) (**b**) in a patient with neurofibromatosis type 1 disease. Lunge: Lung. The origin of the sarcoma remained unresolved. Neurofibromas were also evident in the skin and abdomen.

While we were not successful in finding case reports of CEUS of primary pulmonary sarcoma in humans, there are case series in veterinary medicine on CEUS of pulmonary tumors, including pulmonary sarcomas in dogs and cats [57,58]. However, in the following image sequence, the features of CEUS in a primary sarcoma of the lung are illustrated (Figure 1). CEUS allows for differentiation between the obstructive lung atelectasis (early contrast enhancement via pulmonary artery) and the sarcoma (later centripetal enhancement via systemic arteries). In addition, a neurofibrosarcoma of the lung with relaxation of the diaphragm is presented in Figure 2.

## 3. Pleural Mesothelioma

Pleural mesothelioma is a primary neoplasm of the pleura. Mesothelioma originates from the mesothelium of the pleura, the peritoneum, the pericardium and the tunica vaginalis. Of these various localizations, pleural mesothelioma is the most common, accounting for 90% [59]. Data from the years 2008 to 2012 describe a worldwide standardized incidence rate (WSIR) per 100,000 people of 0.9 for men and 0.3 for women in the USA. In Europe as a whole, the WSIR per 100,000 was 1.7 for men and 0.4 for women. Mesothelioma is extremely rare in younger people, but the incidence then rises significantly between the ages of 50 and 60. At the age ≥80 years, the IR for men in the USA and Europe is 18.9 and 22.8 per 100,000, respectively, and 3.0 and 3.4 per 100,000 for women [60]. It is closely associated with asbestos exposure and can occur after a latency period of up to 40 years following asbestos exposure [61]. Ionizing radiation (mainly therapeutic radiation) is also a risk factor for mesothelioma, but compared to asbestos, significantly fewer people are affected [62].

Mesotheliomas usually originate in the parietal pleura in the inferior hemithorax and costodiaphragmal recessus, causing ipsilateral pleural effusion and spreading into the visceral pleura and surrounding structures of the chest wall, lungs, diaphragm, pericardium or mediastinum.

Common symptoms are dyspnea and chest pain. Dyspnea is a consequence of the pleural effusion or a result of restriction due to extensive diffuse tumors encasing the lungs by solid tumor masses.

The 2021 WHO classification for tumors of the pleura and pericardium [63] differentiates between three major histologic subtypes of mesothelioma (i.e., epithelioid, biphasic and sarcomatoid) and an additional, clinically more indolent “well-differentiated papillary mesothelioma tumor”. Localized mesothelioma is differentiated from diffuse mesothelioma because inoperability is associated with a significantly poorer prognosis. There have been some changes with regard to mesothelioma since the 2015 WHO classification. The prefix “malignant” will be omitted for localized and diffuse mesotheliomas because all mesotheliomas are considered malignant [63].

The recommendations for the management of mesothelioma are summarized in the ERS/ESTS/EACTS/ESTRO guidelines of the European Respiratory Society (ERS)/European Society of Thoracic Surgeons (ESTS)/ European Association for Cardio-Thoracic Surgery (EACTS)/European Society for Radiotherapy and Oncology (ESTRO) [61].

Diffuse or nodular pleural thickening and, in particular, involvement of the mediastinal pleura and especially with a history of asbestos contact are indicative of mesothelioma.

In the ERS/ESTS/EACTS/ESTRO guidelines [61], a diagnostic algorithm was recommended for mesothelioma: After abnormal findings in the X-ray, a CT thorax/abdomen is performed for basic staging in all patients who are in sufficient physical condition for treatment. PET-CT and endosonography/ endobronchial ultrasonography (EUS/EBUS) are performed as part of further staging for all patients for whom surgery is being considered. For patients with borderline resectability prior to radical surgery, additional chest/abdominal ±brain MRI (depending on symptoms), laparoscopy/contralateral video-assisted thoracic surgery and mediastinoscopy are recommended depending on the findings [61].

The gold standard for confirming the diagnosis is histological examination of material obtained from the pleural masses. In the first instance, thoracoscopic sampling is recommended. Image-guided biopsies are an alternative, especially for frail patients who are not fit enough for thoracoscopy. Image-guided needle biopsies are less invasive and have a high diagnostic rate. This applies to patients with suspicious pleural thickening with or without pleural effusion. US-guided biopsy in particular enables a safe biopsy without radiation. Although transthoracic US has the advantages of a very high spatial resolution and real-time-imaging, it has also the important disadvantage of being limited by the bony structures of the chest wall, so that even under optimal conditions only approximately two-thirds of the pleural surface can be depicted [64]. Therefore, in mesothelioma, as in any malignant pleural disease, US must be regarded as a complementary imaging modality.

Despite the existing evidence for US findings in malignant pleural effusion, there is not much literature on mesothelioma-specific US findings. Unilateral pleural effusion is very common in mesotheliomas; in 50 percent of the cases, it is accompanied by an extensive diffuse or nodular pleural thickening [65], with a higher specificity for nodular changes [66].

In a study of 52 patients with pleural effusion, including 33 patients with malignant pleural effusion and 14 patients with mesothelioma, pleural thickening >1 cm and diaphragmatic (nodular) thickening >7 mm were described as signs of malignancy, discriminating benign and malignant pleural diseases with a sensitivity of 79%, specificity of 100%, PPV of 100% and NPV of 73% [67]. In malignancy, pleural thickening was both hypoechoic and iso- or hyperechoic, whereas in benign effusion it was always hypoechoic. Diaphragmatic thickening >7 mm was predominantly a sign of malignancy. Nodular thickening occurred exclusively in malignancy [67]. However, the US signs of the 14 patients with mesothelioma were not specifically reported.

The pleural thickening in mesothelioma is also described as hypoechoic, commonly more extended (>10 mm) than in other pleural diseases and presenting with circumferential growth and/or broad nodular masses [68,69]. An extension into the interlobar fissures must be evaluated.

Pleural plaques are thickenings of the parietal pleura, which consist of connective tissue that can calcify. They are probably the most common radiological manifestation of long-standing asbestos exposure, detected on radiological imaging in about 20% of affected patients with long-term asbestos exposure. On US, they are primarily hypoechoic, but their echogenicity increases with the presence of fibrosis and calcifications. The latter are typically hyperechoic with dorsal sound cancellation [68].

In contrast to these descriptions of US findings in mesothelioma, another study found no specific pathognomonic characteristics for mesothelioma in different imaging modalities [70]. In 80 included patients with suspected mesothelioma, of whom diagnosis was confirmed in 67.5% of the cases, imaging characteristics in direct digital radiography (DDR), US and CT were investigated. Pleural effusion was described in 94.4% of the cases, and non-specific US findings or US-undetectable lesions in 5.6%. Invasion of the thoracic wall was not found in any case. All imaging modalities showed high sensitivity (CT 94.4%, US 92.6%, DDR 90.7%), but poor specificity (DDR 46.2%, CT 35.5%, US 23.8%) [70].

**Figure 3:**
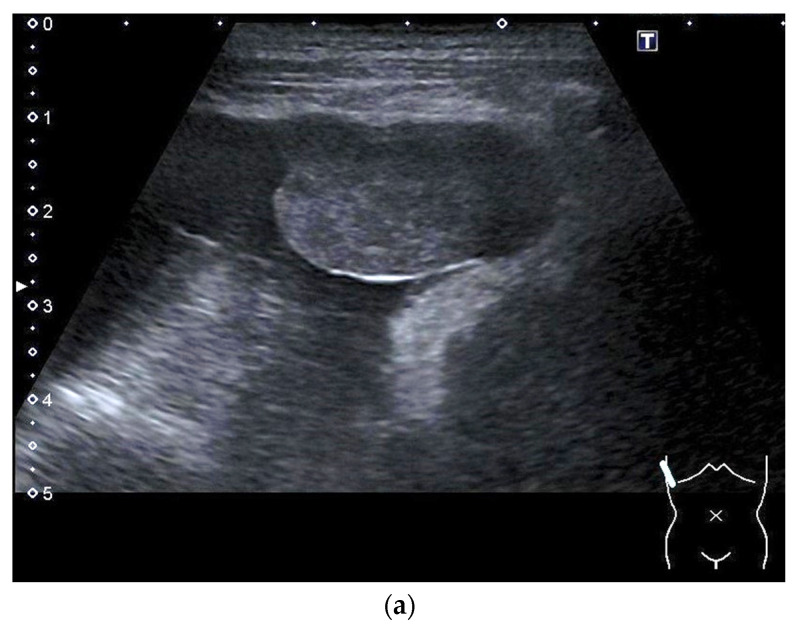

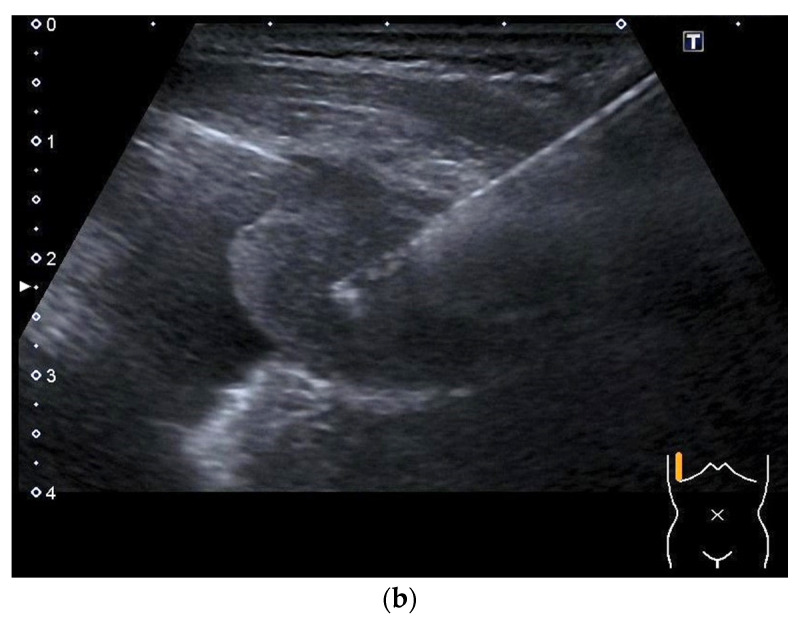
Mesothelioma (epithelioid cell type) in an 81-year-old female patient hospitalized for shortness of breath, vertigo and a weight loss of 10 kg in 6 months. There was a history of breast cancer 15 years ago with complete remission after surgery and adjuvant radiation, but no history of known exposure to asbestos. Ultrasound revealed a large unilateral pleural effusion with a solid tumor at the parietal pleura in the costodiaphragmal recess (**a**). Histology from biopsies confirmed mesothelioma (**b**).

**Figure 4:**
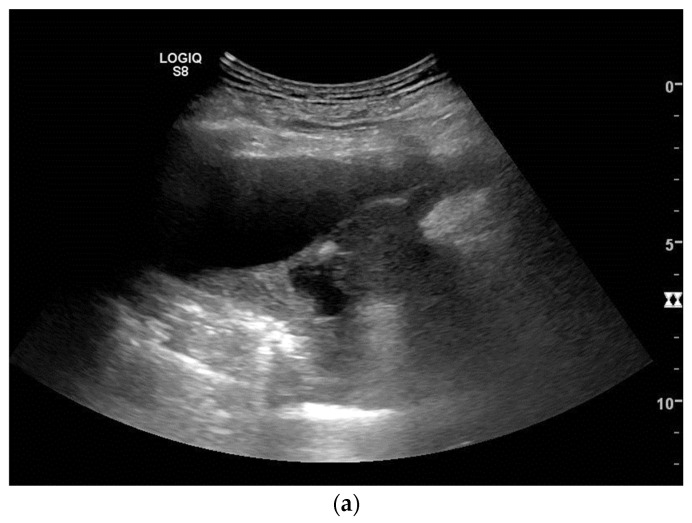

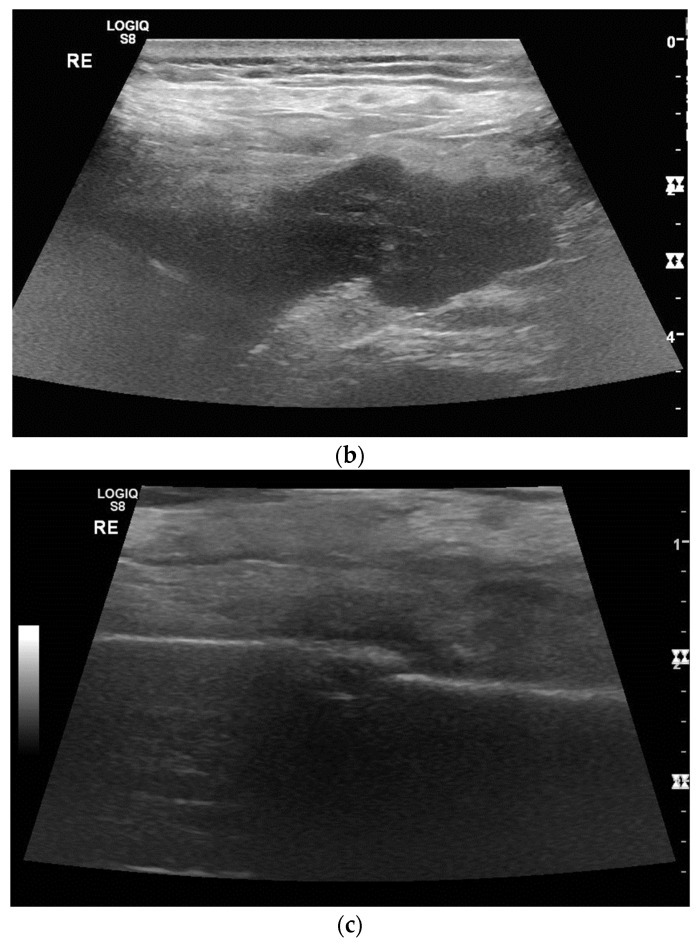
Year-old male patient hospitalized for shortness of breath and located right-sided chest pain. There was no history of asbestos exposure. Ultrasound revealed pleural effusion and tumor masses at the parietal pleura (**a**) with infiltration of the diaphragm and chest wall (**b**) thereby causing a rib fracture (**c**). Histology from biopsies confirmed mesothelioma.

Careful sonographic examination and targeted adjustment of the radiologic imaging findings provided revealed that pleural effusion and thoracic wall or pleural thickening and nodular lesions are again within the scope of US (Figure 3 and Figure 4).

In addition to the description of pleural thickening on B-mode US, CEUS can provide some potential information as to whether malignant pleural thickening is present. However, a specific contrast enhancement of pleural mesothelioma in CEUS has not been described so far.

In a prospective single-center study, B-Mode US and CEUS criteria were investigated in 50 patients with unclear pleural thickening (malignant n = 30, among them 3 mesothelioma cases; benign n = 20). Pleural thickness, separately assessed in B-Mode US and CEUS, was significantly increased in malignant diseases (both *p* < 0.05). In the malignant pleural thickenings, the arrival time (AT) and time to peak (TTP) of the Time Intensity Curve (TIC) were significantly shorter, whereas the peak intensity and the area under the TIC were significantly higher (all *p* < 0.05). Specific CEUS patterns for the three included mesotheliomas were not reported [44].

In studies with CEUS-guided sampling, either no patients with mesothelioma were included or the enhancement pattern was not described for these patients [44,71].

However, Yusuf et al. [72] demonstrate the US and CEUS imaging in figures of a patient with pleural mesothelioma. On US, the mesothelioma appeared as a relatively poorly demarcated lesion. The tumor was difficult to differentiate from the adjacent striated muscles of the thoracic wall. The lesion was hyperenhanced on CEUS, and areas of necrosis were also present. Furthermore, CEUS allowed for a reliable differentiation from the striated muscles of the thoracic wall [72]. Tissue acquisition was performed under CEUS guidance to avoid biopsy of necrosis or the thoracic wall [72].

**Figure 5:**
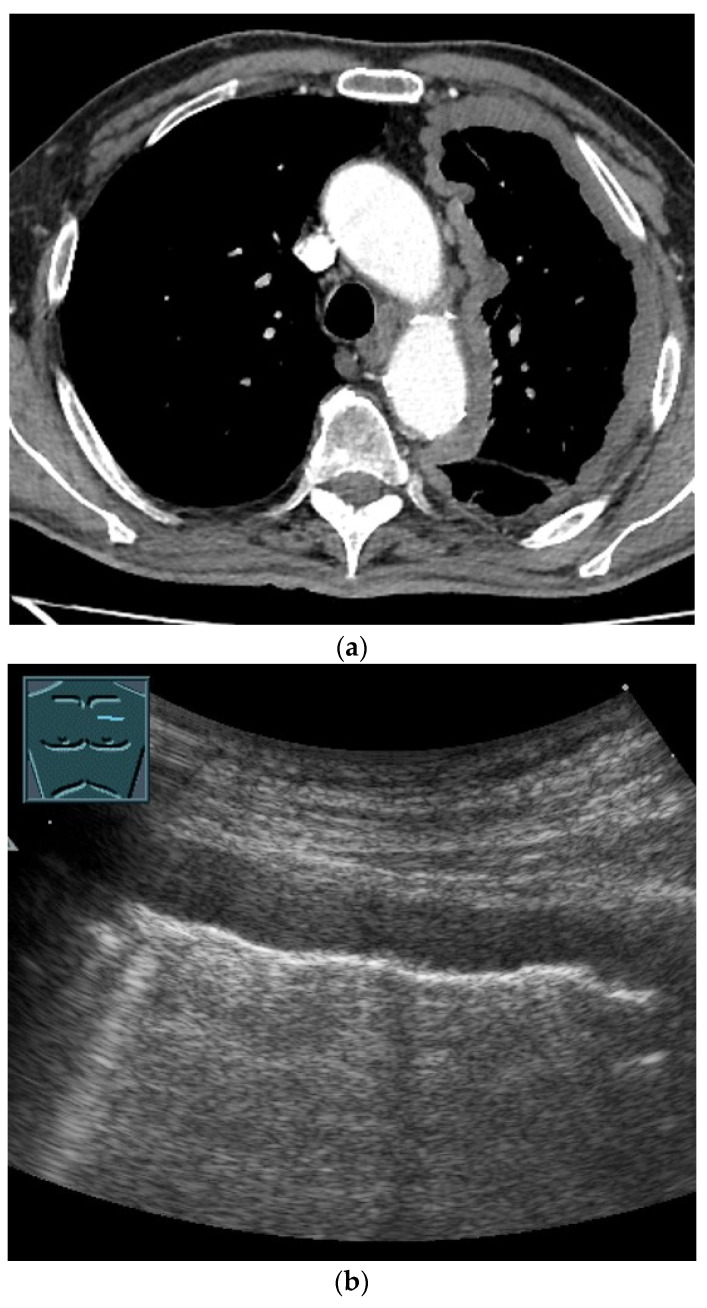

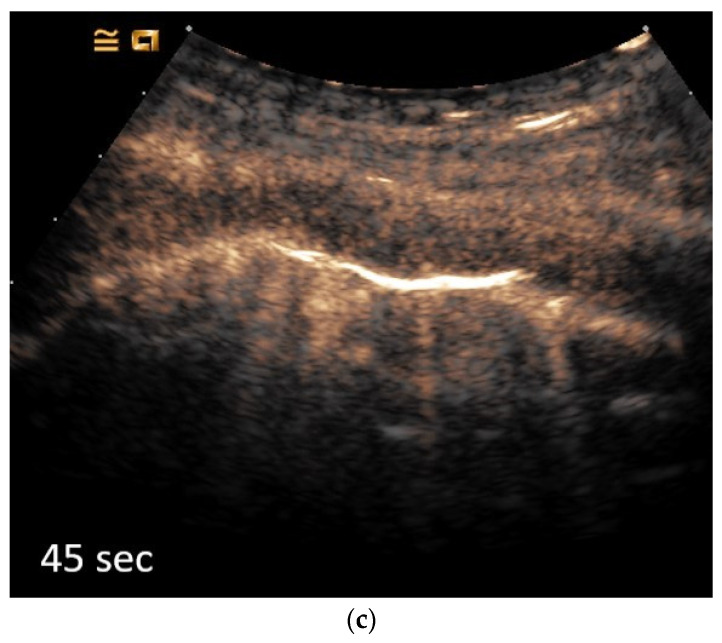
Eighty-year-old patient with a history of asbestos exposure and histologically confirmed epithelioid mesothelioma illustrated on CT (**a**) and B-mode US (**b**). The extensive pleural thickening forming a circular wall around the left lung can be seen on CT (**a**). B-mode US shows a clear hypoechoic thickening of the pleura, which was measured at more than 10 mm (**b**). The lesion is characterized by a moderate systemic arterial enhancement on CEUS (**c**).

**Figure 6:**
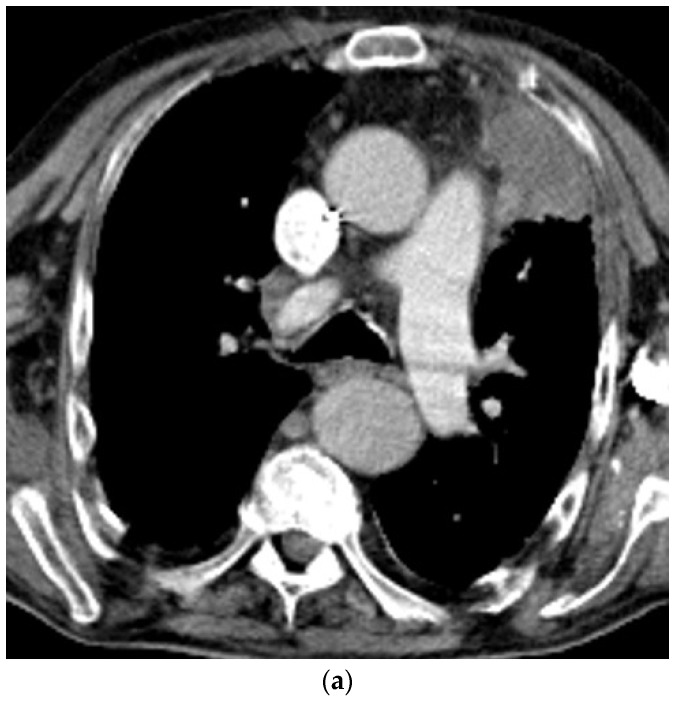

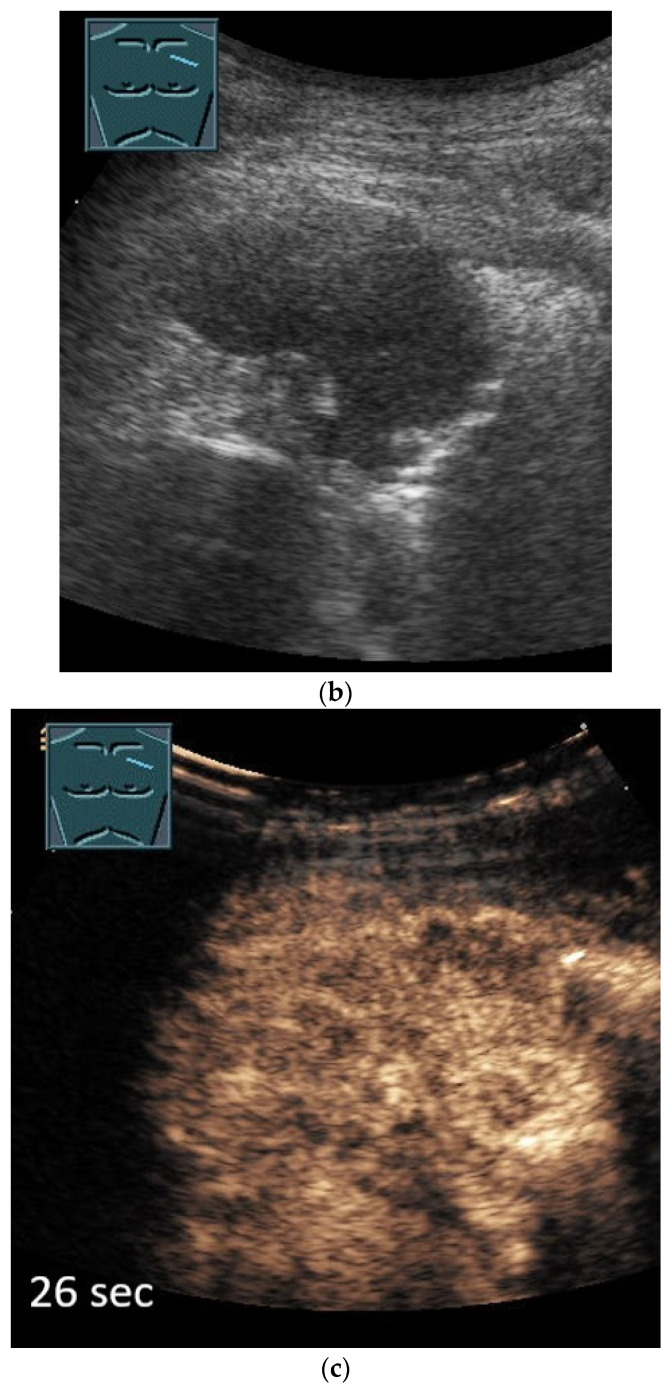

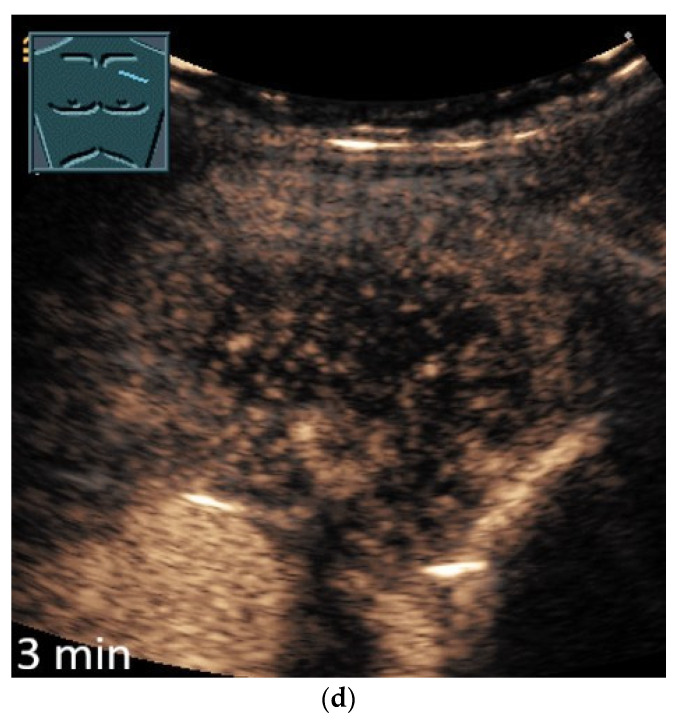
Eighty-year-old patient with histologically confirmed epithelioid mesothelioma shown on CT (**a**) and B-Mode ultrasound (**b**). On CEUS, the lesion shows a marked bronchial arterial enhancement (26 s) (**c**) with parenchymal washout (**d**).

Despite extensive efforts, we were unable to find any further descriptions or illustrations of pleural mesothelioma on CEUS. Our own experience with CEUS in two mesothelioma cases showed arterial enhancement and parenchymal washout in the CEUS (Figure 5 and Figure 6).

The gold standard for confirming the diagnosis is defined as histological material obtained from the pleural masses. Thoracoscopic material collection is recommended in the first instance as this procedure offers better possibilities for macroscopic inspection, generous sampling, hemostasis and, if necessary, talcum pleurodesis. US-guided biopsies are an alternative, especially for frail patients who are not fit enough for thoracoscopy.

Iadevaia et al. investigated whether ultrasound-guided percutaneous needle biopsy (US-PPNB) has a high diagnostic accuracy in the confirmation of pleural mesothelioma and whether it is a safe option for the diagnosis of pleural mesothelioma. The US-guided sampling was performed by an experienced pulmonologist using an 18 G biopsy system. The US-guided biopsy was able to diagnose mesothelioma in 83% of patients (sensitivity: 83.39%; specificity: 100%; PPV: 100%). There was a significant difference in the average thickness of the pleural lesion between patients with adequate and inadequate biopsy (15.4 mm (SD: 9.19 mm) and 3.77 mm (SD: 0.60 mm), *p* < 0.0010 [73]. The diagnostic accuracy was dependent on the size of the pleural lesion [73].

**Figure 7:**
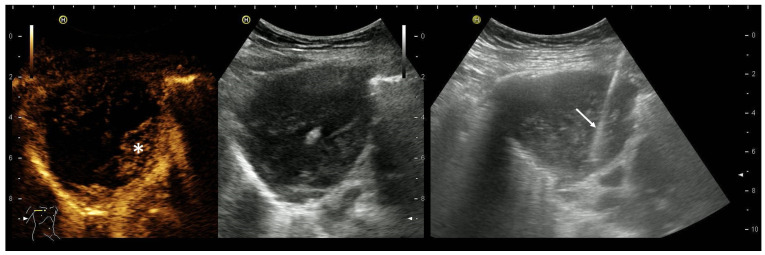
CEUS-guided biopsy of a subpleural lung tumor of a 66-year-old female patient. CEUS revealed large non-enhancing areas; only a small peripheral area was enhancing in the arterial phase (*). The tip of the biopsy needle (arrow) was positioned in this vital part of the tumor to facilitate the procurement of an adequate sample.

Malignant pleural thickening, nodular pleural lesions and thoracic wall-adjacent peripheral pulmonal tumors usually do not have specific characteristics for different tumor entities [74], so that tissue acquisition for histology and immunohistochemistry using US guidance should be performed [35,75,76]. CEUS can be used to visualize architectural features, delineate necroses and determine the most appropriate access route. Moreover, it helps delineating small pleural and subpleural pulmonary lesions and differentiating necrosis from vital tumor tissue, thereby decreasing the risk of false-negative results [33,35,71,77,78,79,80] (Figure 7).

**Figure 8:**
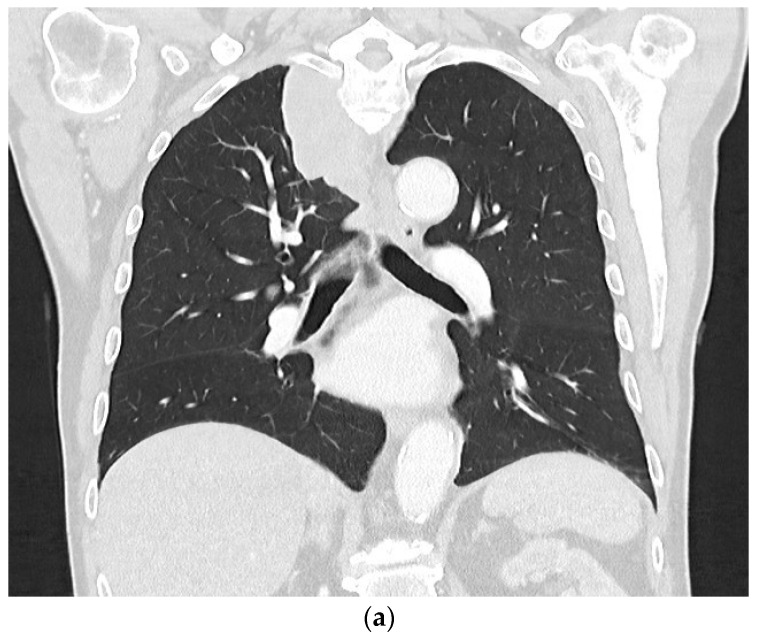

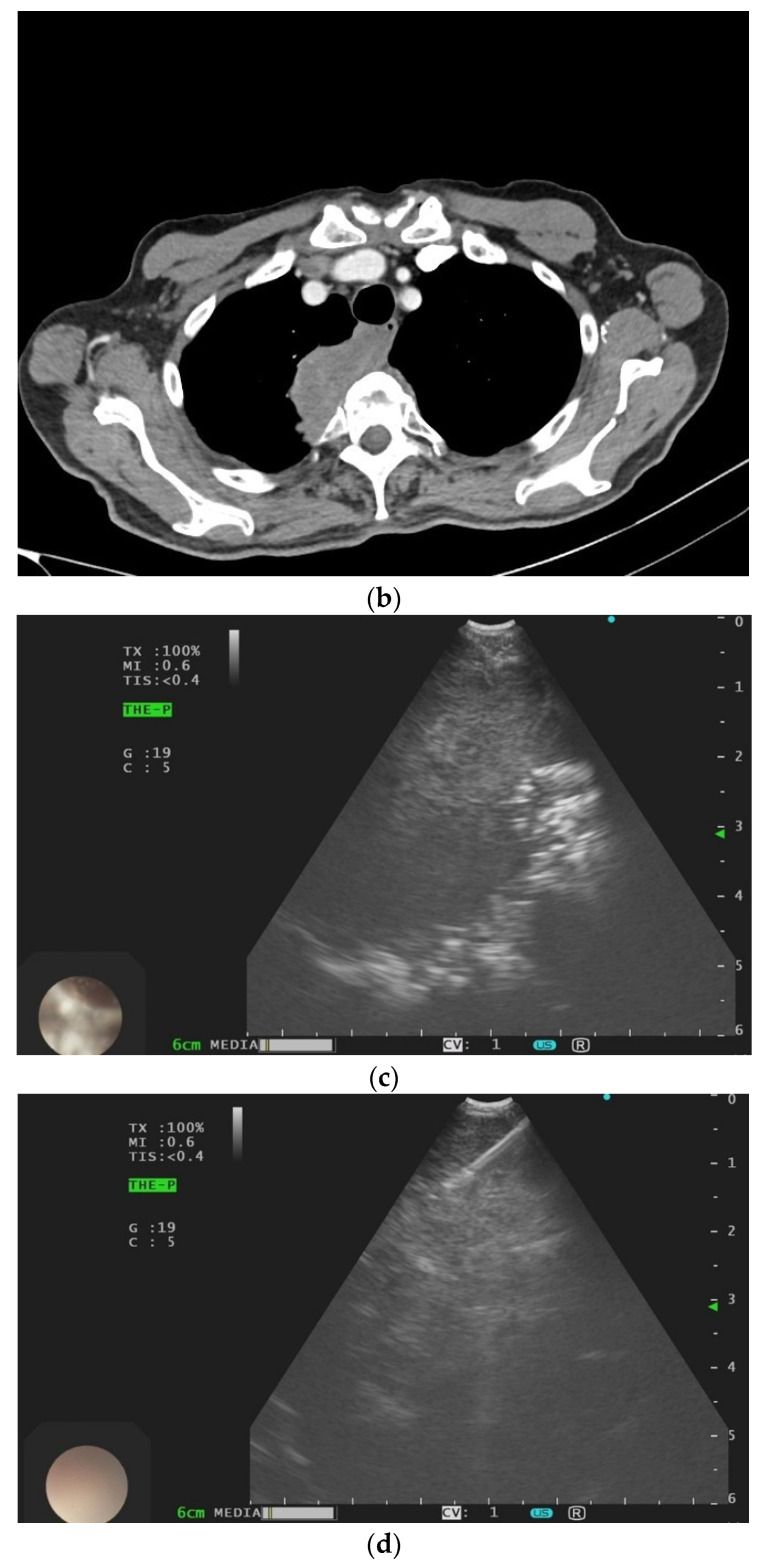
A male patient reported asbestos exposure about 45 years ago. CT imaging revealed a tumor in the mediastinal pleura of the right upper thorax (**a**,**b**). EUS performed transesophageally with an EBUS bronchoscope (Olympus) (EUS-B) showed a solid inhomogeneous tumor and adjacent compressed lung tissue (**c**). The performance of an EUS-B-FNB (Mediglobe Top-Gain 22 G) confirmed an epithelioid mesothelioma (**d**).

If the tumor masses are adjacent to the esophagus, trachea or bronchi, they can also be characterized using EUS- or EBUS-guided sampling (Figure 8).

The treatment concept is aimed at multimodal treatment including macroscopic complete resection (only in expert centers). In case of local or functional inoperability, therapeutic alternatives consist of palliative chemotherapy (standard first-line: platinum/pemetrexed), radio-chemotherapy or best supportive care [61].

## 4. Conclusions

Pulmonary findings that are adjacent to the pleura and are not overlaid by lung artifacts can be detected and described by US.

Primary pulmonary sarcomas are very rare, and reports on the US features of these often very large tumors are almost lacking. With the exception of the case presented in this article, reports on characterization with CEUS are only available from veterinary medicine.

Pleural mesothelioma is characterized by a thickening of the pleura, which can surround the entire lung and also extend into the interlobular space. In the case of extensive findings and especially if there is a history of contact with asbestos, these changes suggest a diagnosis of mesothelioma. However, the diagnosis has to be confirmed histologically. Ultrasound-guided sampling is an alternative to thoracoscopic biopsy in frail patients. CEUS can be used to better delineate the tumor and differentiate vital tissue components from necrosis and thus achieve more adequate tissue samples. The data presented give rise to a greater use of transthoracic US in the diagnosis of lung and pleural tumors in addition to CT. CEUS in particular provides additional possibilities for characterization and targeted tissue acquisition.

## Data Availability

The data presented in this study are available on request from the corresponding author. The data are not publicly accessible for the following reasons, as the personal rights of the patients concerned must be respected.
